# Genomic and transcriptomic analysis of the toluene degrading black yeast Cladophialophora immunda

**DOI:** 10.1038/s41598-017-11807-8

**Published:** 2017-09-12

**Authors:** Barbara Blasi, Hakim Tafer, Christina Kustor, Caroline Poyntner, Ksenija Lopandic, Katja Sterflinger

**Affiliations:** Department of Biotechnology, VIBT-EQ Extremophile Center, University of Natural Resources and Life Sciences, 1190 Vienna, Austria

## Abstract

*Cladophialophora immunda* is an ascomycotal species belonging to the group of the black yeasts. These fungi have a thick and melanized cell wall and other physiological adaptations that allows them to cope with several extreme physical and chemical conditions. Member of the group can colonize some of the most extremophilic environments on Earth. *Cladophialophora immunda* together with a few other species of the order Chaetothyriales show a special association with hydrocarbon polluted environments. The finding that the fungus is able to completely mineralize toluene makes it an interesting candidate for bioremediation purposes. The present study is the first transcriptomic investigation of a fungus grown in presence of toluene as sole carbon and energy source. We could observe the activation of genes involved in toluene degradatation and several stress response mechanisms which allowed the fungus to survive the toluene exposure. The thorough comparative genomics analysis allowed us to identify several events of horizontal gene transfer between bacteria and *Cladophialophora immunda* and unveil toluene degradation steps that were previously reported in bacteria. The work presented here aims to give new insights into the ecology of *Cladophialophora immunda* and its adaptation strategies to hydrocarbon polluted environments.

## Introduction

Aromatic compounds like benzene, toluene, ethylbenzene and xylene isomers, collectively known as BTEX, are one of the major contributors to environmental pollution, globally threatening natural environments, groundwater reservoirs^1, 2^, agricultural sustainability and, as a consequence, food safety. They naturally occur in coal, oil and gas deposit and are also one of the most abundantly produced chemicals in the world. Their release into the environment happens through natural events, like forest fires or volcanic eruptions, and human activities, such as vehicle traffic and the coal, gas and oil exploitation chain. The majority of BTEX are released into the atmosphere, but can also accumulate in ground waters and soils located close to natural and anthropogenic BTEX sources^1, 2^.

Long-term BTEX exposure can have deleterious effects on the liver, kidneys, central nervous system, lungs and cause endocrine disruption^3^. As a consequence, the production and release of BTEX are strongly regulated by national, supranational and international policies. While technologies for air pollution control or soil remediation are available, these compounds are difficult to remove, since they remain toxic at concentration levels where chemical or physical removal is not economically sustainable^4^. Promising and cost-effective alternative to physico-chemical environmental remediation are slowly emerging. In case of air pollution control, biofilter, where bacterial or fungal communities degrade pollutants from contaminated gases, gained a lot of attention^5^. Even though bacteria-based biofilter are more widely used^6^, fungal biofilters are more efficient upon dehydration and acidification of the media^7, 8^.

The most extensively studied fungal isolates in the field of bioremediation belong to the genera *Exophiala*, *Cladophialophora*, *Aspergillus*, *Phanerochaete*, *Cladosporium*, *Paecilomyces*, *Trichoderma* and *Trametes* ^9–12^. In recent decades the group of the black yeasts, where Exophiala and Cladophialophora are classified, have begun to be recognized for their bioremediation potential^12^. Some of these fungi have in fact been isolated from different polluted sources^13^, such as industrial spills, car gasoline tanks, railway sleepers^14^ and air biofilters^15^. The common characteristic of these fungal group members is the presence of melanin, constitutively expressed and deposited at the cell wall level. In particular, the genera *Exophiala* and *Cladophialophora* (order Chaetothyriales), and *Pseudallescheria* (order Microascales) have a high potential to grow in polluted environments and to metabolize hydrocarbons as the sole source of carbon and energy^12^. Especially *Cladophialophora immunda* was characterized for its ability to degrade up to 65% of the toluene supplied^12^.

Although most of the known *Cladophialophora immunda* strains were sampled from environmental polluted sources, the fungus is also an opportunistic human pathogen, causing subcutanoeus phaeohyphomycosis in immunosuppressed patients^16^. It is currently hypothesized that this dual ecology may arise from the hydrocarbon degrading pathways, which would allow the fungus to use the neurotransmitters for their own energy metabolism^17^. While light has been shed on the mechanisms behind black yeasts ecology and pathogenicity^18–20^, the biological processes responsible for the xenobiotics-degrading ability of these fungi have not yet been studied with next-generation sequencing approaches. In order to get an insight into the mechanisms of toluene tolerance and degradation, we present the first genomic and transcriptomic analysis of *Cladophialophora immunda* upon growth with toluene as sole carbon and energy source.

The strain used in this experiment (CBS 110551) has been isolated from an industrial biofilter operated with toluene air stream that was previously inoculated with soil from a gasoline station^13^. The fungus was inoculated in liquid mineral media and exposed to vapors of toluene through hand-made test tubes in sealed Erlenmeyer flask. In the control experiment, the fungus was grown in the same media with 2% glucose as classical carbon source for one week. At the end of the incubation times, the biomass was collected through filtration and used for RNA isolation and sequencing.

We performed a comparative genomics analysis of *Cladophialophora immunda* with other toluene-degrading and pathogenic species. Cytochrome P450 and fungal specific transcription factors were among the most overrepresented domains compared to the other genomes. Genes horizontally transferred from bacteria were found to play an important role in *C*. *immunda* toluene degradation pathway. The transcriptome analysis revealed that in this fungus toluene triggers the expression of genes involved in the putative fungal toluene degradation pathway, oxidative stress as well as DNA repairs and molecular chaperones.

## Method

### Fungal growth conditions

*Cladophialophora immunda* (CBS 110551), a strain isolated from a toluene-charged air biofilter inoculated with gasoline-polluted soil, was ordered from Centraalbureau voor Schimmelcultures. The fungus was cultured in Malt extract agarose media (2% Malt extract, 2% D-glucose, 0.1% Bacto-peptone and 2% Agar). For the RNA-seq experiments, *Cladophialophora immunda* was grown in liquid culture in a modified Hartmans mineral^21^ media with 2% glucose or 1.35 mM toluene as sole carbon source, 1 ml of vitamin solution and 0.02% of yeast extract as nitrogen source and to boost the fungal growth by shortening the lag phase^22^. For the exact recipe of the mineral media and vitamin solution consult the Supplementary materials. The toluene was supplied through the air of a sealed flask in a 5% solution in dibutylphtalate. Depending on the carbon source, the experiments duration was set to 90 days for toluene and one week for glucose, both at temperature of 22 °C and 100 rpm on an orbital shaker. Both experiments were performed in triplicates. At the end of the experiments the biomass was collected by centrifugation (5000 g per 15 minutes at 4 °C), washed with RNAse free water, frozen in liquid nitrogen and stored at −80 °C until use.

### RNA isolation and sequencing

Total RNA was extracted from 100 mg of fungal biomass with FastRNA PRO^TM^ RED KIT (MP Biomedicals) according to the manufacturer instructions. The mRNA was isolated with the Dynabeads^©^mRNA DIRECT^TM^ Micro Kit (Ambion by Life Technologies) and the subsequent transcriptome library preparation was performed with the Ion Total RNA-Seq Kit v2 (Life Technologies). Total RNA, isolated mRNA and the final cDNA libraries were all qualitatively and quantitatively evaluated with the Agilent 2100 Bioanalyzer (Agilent Technologies, Santa Clara, CA). The transcriptome sequencing was performed by the Ion Proton^TM^ sequencer (Life Technologies) with the Ion PI Chip v2 (Life Technologies).

### Bioinformatics

#### Genome annotation

The genome sequence used was previously published in ref. 23. The denovo non-coding RNA (ncRNA) annotation was done with Infernal^24^ and the Rfam database^25^, tRNAscan^26^ and SNOSTRIP^27^. Overlapping ncRNAs annotations were merged manually.

For the protein coding genes, the reads from all 6 RNA sequencing runs were assembled with Trinity 2.0.6^28^ and Cufflinks 2.2.1^29^. Both sets of transcripts were merged with the help of PASA^30^. Transdecoder^30^ was then used to predict protein coding transcripts. Intron/Exon from these coding transcripts were used to train Augustus^31^. The prediction from Augustus and PASA were subsequently merged into a set of protein coding loci with EvidenceModeler^32^.

PASA-Transcripts that were not considered coding by Transdecoder and by CPAT^33^ (p-value  > 0.01), that did not show significant (p-value > 0.001) sequence homology against SWISSPROT when searched with Blastn^34^ and that did not contain Pfam-domain^35^ (p-value > 0.001) when searched with HMMER^36^ were classified as non-coding. The transcripts that could not be unequivocaly classified as either coding or non-coding were classified as transcript with unknown functions. Samtools^37^ were used to process the mapped reads. Bedtools^38^ were used to look for annotation overlaps.

Finally conserved coding and non-coding elements were detected with RNAcode^39^ (p-value < 0.01) and RNAz^40^ (probability P > 0.9), respectively. To this aim a multiple genomes alignment of *Aspergillus niger*, *Aspergillus nidulans*, *Aspergillus fumigatus*, *Cladophialophora immunda*, *Exophiala dermatitidis*, *Fusarium oxysporum*, *Fusarium solani*, *Neurospora crassa*, *Saccharomyces cerevisiae*, *Schizosaccharomyces pombe* and *Trichophyton rubrum* was generated with the multiz pipeline^41^ implemented in snakemake^42^.

#### Comparative genomics

Gene sequences and annotations for *Aspergillus niger*, *Aspergillus nidulans*, *Aspergillus fumigatus*, *Cladophialophora bantiana*, *Exophiala dermatitidis*, *Fusarium oxysporum*, *Fusarium solani*, *Neurospora crassa*, *Saccharomyces cerevisiae*, *Schizosaccharomyces pombe* and *Trichophyton rubrum* were fetched from ensembl and ncbi. For *Exophiala mesophila*, our own genome assembly and annotation were used^43^.

The protein-coding genes of all studied species were functionally annotated with a local install of interproscan-5.19–58.0^44^ with an E-value cut-off of 1.10^−3^. Homologies with the transporter classification database (TCDB)^45^, the peptidase database (MEROPS)^46^ and the carbohydrate-active enzymes database (CAZY)^47^ were assessed with Blastp^48^ (E-value < 1e-3). Genes homology was assessed with Proteinortho-5.13^49^. Horizontally transferred genes were detected with the latest version of HGTFinder (2016)^50^.

#### Genes expression

All reads were mapped with STAR 2.5.0^51^ with – – *alignIntronMin*15 – *alignIntronMax*2000 – *chimSegmentMin*12 – *chimJunctionOverhangMin*12 – *alignSJDBoverhangMin*10 against the *Cladophialophora immunda* genome. The mapped reads were then used to find differentially expressed genes and detect chimeric RNAs. The raw and mapped reads were used to assemble the transcriptome of *Cladophialophora immunda*. Count of mapped reads on annotation elements was done with featureCounts v1.4.6p2^52^. The identification of differentially expressed genes was done with edgeR^53^, while the functional enrichment of the significantly regulated genes was done with GoStat^54^ and Kobas^55^. Revigo^56^ was used to summarize the list of Gene Ontology terms. Expression level analysis was done with Salmon 0.7.1^57^ with 30 bootstraps, sleuth and wasabi.

## Results

### Highly complete genome and genes set

The genome assembly of *Cladophialophora immunda* used for this study was previously published by our group^23^. It has a size of 42 Mb and includes 464 contigs (>500 bp). A total of 15228 protein-coding genes (17887 proteins) were predicted with a combination of gene annotation tools using sequence homology and strand-specific sequencing of RNA (RNAseq, see Method). 15214 were supported by RNAseq (TPM > = 0). 95.56% of ultra-conserved genes found in CEGMA^58^, were recovered in *Cladophialophora immunda*. Among the basal small RNAs, 52 tRNAs (28 spliced), 46 snoRNAs, all snRNAs of the major spliceosome as well as RNAse-P, RNAse-MRP, 5S and U3 were found (See Methods). Another isolate of the same species of *Cladophialophora immunda* (CBS 83496) was recently sequenced and contains 12879 genes (14033 proteins)^59^. From the 3854 supplementary proteins in *Cladophialophora immunda* (CBS110551), 1815 had no homologues in CBS 83496 at an E-value threshold of 1.10^−3^. Among these genes, 964 had homologues in the non-redundant protein sequences database (nr), resulting in 851 genes being specific to *Cladophialophora immunda* (CBS110551). Eukaryotic rRNA sequences were found on contig JSEJ01000356, while annotation corresponding to mitochondrial tRNAs and rRNAs were detected mainly on contigs JSEJ01000378 and JSEJ01000388 (See Supplementary Table S1).

A total of 5300 non-coding transcripts (lncRNAs) from 4507 genomic loci were assembled. 10178 transcripts with a non-negligeable protein-coding potential and coming from 9301 loci were assembled.

The set of conserved coding and non-coding elements were detected with RNAz and RNAcode^39, 40, 60^ on a multiple genomes alignment containing *Cladophialophora immunda* with 10 other fungal genomes (*Aspergillus fumigatus*, *Aspergillus nidulans*, *Aspergillus niger*, *Exophiala dermatitidis*, *Fusarium oxysporum*, *Fusarium solani*, *Neurospora crassa*, *Saccharomyces cerevisiae*, *Schizosaccharomyces pombe*, *Fusarium solani*). The resulting coverage of the *Cladophialophora immunda* genome ranged from 4.8% for *Saccharomyces cerevisiae* to 39.6% for *Exophiala dermatitidis*. The *Cladophialophora immunda* -centered multiple alignment featured a coverage of 79% of the fungal genome. RNAz returned a total of 852 structurally conserved regions with a P-score > 0.9, while RNAcode detected 49508 genomic regions with conserved coding potentials (p-value < 0.01) 122 RNAcode regions overlapped with 117 RNAz loci. Non-coding PASA transcripts overlapped with 111 RNAz loci and 44 Rfam candidates, among them tRNAs, snRNAs, snoRNAs or rRNAs.

### Genes conservation and gene family evolution

**Figure 1:**
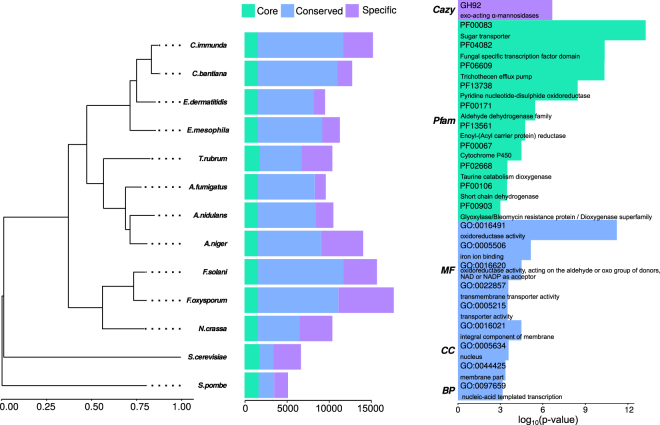
Phylogenetic relationship, gene conservation of *Cladophialophora immunda* compared to 12 other genomes and functional enrichment of *Cladophialophora immunda*-specific paralogues. l.h.s Phylogenetic tree constructed based on the concatenated mafft alignment of 717 one-to-one paralogues. Species phylogeny was inferred with iqtree using genes with one-to-one relationships across all 13 genomes and with 1000 bootstraps. center The orthologue conservation for all 13 species studied is shown. Core genes, i.e. genes found in all genomes, are shown in dark blue. The number of conserved genes, i.e. genes found in at least two species, is shown in blue, while species specific genes are shown in light-blue. r.h.s Barplot of the log_10_(*fdr*) of significantly (fdr <0.001) enriched protein domains, GO terms and Cazy families for the *Cladophialophora immunda*-specific paralogues.

The gene conservation pattern between *Cladophialophora immunda*, the 10 genomes used for the multiple genomes alignments, *Cladophialophora bantiana* and *Exophiala mesophila* was studied with Proteinortho^49^ (see Methods). 1191 genes had homologues in all 13 species, 9742 genes had at least one homologue in any of the species and 5491 genes were specific to *Cladophialophora immunda* (See Fig. 1). 385 paralogues groups, corresponding to 796 genes, were found to be specific to *Cladophialophora immunda*. The significantly enriched protein domains of the *Cladophialophora immunda* -specific paralogues were related to families of transporters, transcription factors, trichothecene efflux pump, cytochrome P450, taurine dioxygenase and short chain dehydrogenase. GO terms were enriched in oxidoreductase activity, iron ion binding, transporter activity, nucleic-acid templated transcription, membrane part and nucleus (see Fig. 1 and Supplementary Table S1). The phylogenetic tree obtained from the alignment of the 771 one-to-one paralogues of 13 fungal species was consistent with the taxonomic tree (See Fig. 1).

The enrichment or depletion of Cazy, Merops and Pfam protein domains in *Cladophialophora immunda* against the other 12 genomes was studied. According to Cazy, the most overrepresented protein domains related to carbohydrates metabolisms belong to two families of glycoside hydrolases: GH16 and GH92 (both with fdr <0.001). Families GH16 and GH92 are glucanase/galactosidases and exo-acting *α*-mannosidases, respectively.

**Figure 2:**
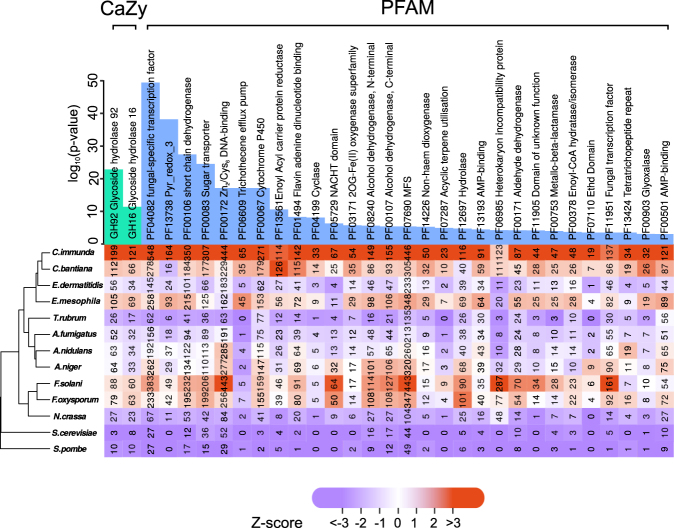
Plot of the overrepresented functional annotations in *Cladophialophora immunda* compared to 12 other genomes. The upper part of the figure shows the fdr of the overrepresented categories as bars in log_10_ scale. PFAM categories are in blue, while CaZy categories are in turquoise. For each annotation element, the number of genes annotated with the categories in each genome is shown. Purple indicates a depletion with respect to the average number of annotated element found in the 13 genomes, while oranges represents an enrichment with respect to the average. The shade of each entry is proportional to the deviation from the average number of annotation for the 13 genomes.

29 Pfam terms were overrepresented, with six Pfam families related to oxygenase, dehydrogenase, transcription, AMP-binding, *β*-oxidation and transport (See Fig. 2). Interestingly, *Cladophialophora immunda* exhibits 65 trichothecene efflux pumps, a much higher number than what is found in fungi like *Fusarium oxysporum* that metabolize this compound^61^. This is also seen in two other black yeasts, *Exophiala mesophila*, an aromatic compounds degrader, and *Cladophialophora bantiana*, a lethal neurotropic pathogen^12, 17^. In fact, a recent publication^59^ showed that trichothecene efflux pumps probably expanded in the precursor of the black yeast group. Further enriched protein families were cyclase, acyclic terpene utilization, hydrolase, heterokaryon incompatibility protein, protein with domain of unknown function, metallo-beta-lactamase and proteins having tetratrichopeptide repeats.

In order to better understand which specific genes might be responsible for the toluene degradation activity of *Cladophialophora immunda* we looked at which genes were specific to *Cladophialophora immunda*, *Cladophialophora bantiana*, *Exophiala mesophila*, *Fusarium oxysporum* and *Fusarium solani* which are, on the exception of *C*. *bantiana*, all (poly-)aromatic degraders^17^. A total of 64 genes were found to be specific to these 5 species (See supplementary Table S1). In *Cladophialophora immunda* these genes were enriched in 30 Pfam families with an *fdr*<0.1. Among them PF14832 (fdr = 0.04), 4-Oxalocrotonate tautomerase, is interesting in the context of toluene degradation, as it was shown to be part of a bacterial metabolic pathway that oxidatively catabolizes toluene, o-xylene, 3-ethyltoluene, and 1,2,4-trimethylbenzene into intermediates of the citric acid cycle^62^.

### Horizontal gene transfer

HGTFinder^50^ was used to look for horizontally transferred bacterial genes. 109 genes in *Cladophialophora immunda* were found to be horizontally transferred from bacteria (q-value  < 0.001) (See Supplementary Table 1). Functional enrichment analysis on this set of genes indicates that they are preferentially involved in carboxylic acid metabolism, hydrolase activity, pectine and cellulose degradation as well as reductase activity (See Supplementary Table S1).

The occurrence of clusters of horizontally transferred genes was also studied. Two genes were considered to belong to the same cluster if they were separated by at most 4 genes. A total of 10 gene clusters were found to be horizontally transferred from bacteria. Gene name, function and q-value of transfer (qT) are listed in Supplementary Table S1.

### RNA sequencing of *Cladophialophora immunda*

The transcriptional landscape of *Cladophialophora immunda* was assessed during growth with either glucose or toluene as sole carbon source. Both experiments were sequenced in triplicates on the Ion Proton platform, yielding a total of 496 million reads with an average length per run between 130 and 159 nucleotides. The mapping rate, depending on the sequencing run, ranged between 50% and 83% of the reads, yielding a total of 320 million reads mapped (See Supplementary Table S1 for more details). The majority of the unassigned reads could be mapped against rRNAs, indicating that the poly(A) enrichment protocol did not completely discard rRNAs. Despite the rRNA contamination, sample expression similarity assessed with principal component analysis fits well with the expectation from the experimental design (See Supplementary Figure S1).

Functional enrichment for the 100 most highly expressed coding genes in the toluene and glucose samples was analyzed. In the glucose experiment, genes related to translation (fdr = 9.87 · 10^−15^), ion transport (fdr = 3.69 · 10^−5^) and energy metabolism (fdr = 1.64·10^−5^) are overrepresented, while in the toluene experiment, genes involved in translation initiation (fdr = 5.05 · 10^−5^) and protein folding (HSP20, ClpA/B, HSP40, HSP70) are overrepresented.

#### Differential expression

The differential expression of Rfam annotated ncRNAs in *Cladophialophora immunda* were computed in the toluene experiment compared to the glucose experiment. 15 ncRNAs, exclusively snoRNAs, were upregulated with a fdr <1.00 · 10^−3^ in the toluene experiment. On the other hand, 17 tRNAs, 1 snRNA (U6), 10 5S rRNAs and SRP were downregulated in the toluene experiment. Among the assembled non-coding transcripts, 268 were upregulated and 199 were downregulated with a fdr <1.00 · 10^−3^.

**Figure 3:**
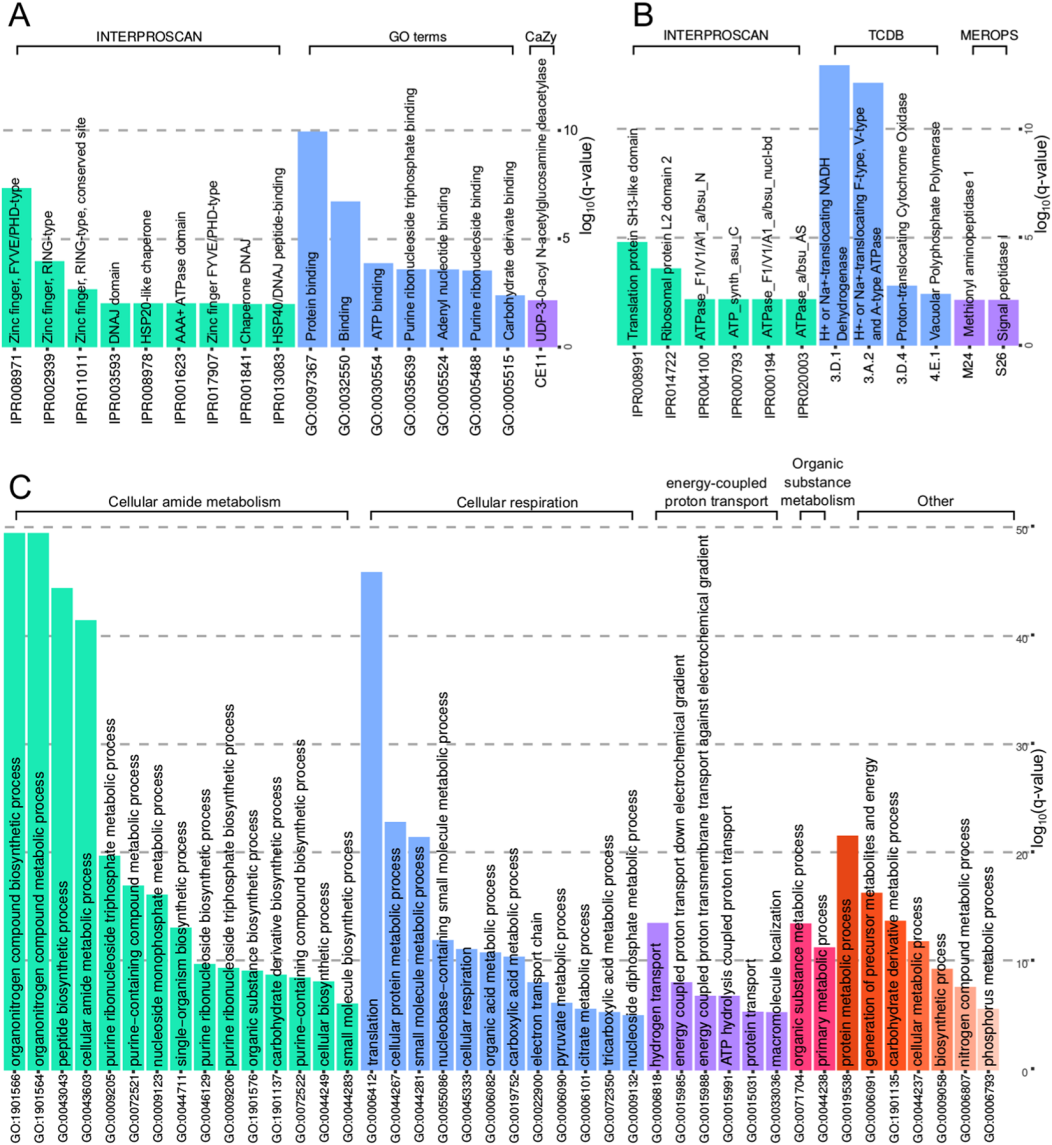
Functional and protein domain enrichment analysis of the genes significantly regulated between the toluene and glucose experiment. The height of each bar represents the *log*_10_ of the fdr of the enrichment of each category. (**A**) Interproscan, Cazy and GO terms enrichment for the genes upregulated in the toluene experiment. (**B**) Protein domain enrichment for the downregulated genes. (**C**) GO term enrichment for the downregulated genes. The GO terms were clustered with REVIGO. GO terms with the same color belong to the same cluster. The most representative term of each cluster is on the top. For the cluster named *Other*, each bar represents a cluster of its own.

543 and 355 protein-coding genes were up- and downregulated (fdr <1.00 · 10^−3^, $|log2FC\mathrm{| > 3}$), respectively between the toluene and glucose experiments. In the set of upregulated genes, protein domains linked to protein folding (HSP40, DNAJ, HSP20, cis-trans isomerase, DSBA-like thioredoxin)^63^ and protein-protein interactions (Ring-Type Zing finger) are overrepresented (See Fig. 3 and Supplementary Table S1). Further 11 out of 47 proteins involved in the transport of misfolded proteins (TCDB family 3.A.16, fdr = 6.83 · 10^−2^) and 5 out of 16 genes related to damaged DNA binding (GO:0003684, fdr = 3.84 · 10^−2^) were upregulated in the toluene experiment (See Supplementary Table S1). Genes involved in the degradation of xenobiotics and detoxification processes^64^ like Glutathione S-transferase (CLAIMM_13540 *x*96, CLAIMM_02727 *x*4), CLAIMM_13949 (*x*28, Glyoxylase II), glutathione-dependent formaldehyde-activating enzyme (CLAIMM_10595 *x*51, CLAIMM_02133 *x*12, CLAIMM_10984 *x*15) are also upregulated in the toluene experiment. The expression of two antioxidants, ascorbate peroxidase (CLAIMM_03119 *x*12) and carotenoid oxygenase (CLAIMM_10918 *x*12) was also triggered when *Cladophialophora immunda* is exposed to toluene.

The set of downregulated genes covers a broader spectrum of functions than that of the upregulated genes. The GO terms enriched can be roughly grouped into amide metabolism, cellular respiration, proton transport and translation (See Fig. 3 and Supplementary Table S1).

At a cellular level, one of the main effect of toluene is the increase in membrane fluidity, which can cause leakage and damages of proteins and cellular organelles, as observed in bacteria^65^. For this reason, the presence of significantly regulated genes related to cell-wall biosynthesis and lipid metabolism was investigated. From the 64 genes that are homologues to cell-wall biosynthesis genes in *Exophiala dermatitidis* ^18^, 16 are downregulated in the toluene experiment while one is upregulated (See Supplementary Table S1). The repressed genes are involved in chitin synthase, regulation, modification, degradation as well as 1.3-*β*-glucan synthesis and processing. The fungal ergosterol biosynthetic pathway exhibit three downregulated genes (Erg2, Erg5, Erg10) while Erg12, mevalonate kinase, is upregulated (See Supplementary Table S1). Among the 11 genes related to membrane lipids metabolisms (e.g. di- and tri- acyl glicerols synthesis), two exhibited a significantly increase in expression, while none were downregulated (See Supplementary Table S1).

### Potential for secondary metabolites

A total of 152 distinct secondary metabolite biosynthesis gene clusters (SMBGCs) were predicted by antiSMASH^66^ and SMURF^67^. antiSMASH reported that 145 gene clusters had similarity in other species and 8 gene clusters were similar to known SMBGCs (See Supplementary Table 1 and Table 1). Monacoline K is the product of the SMBGC with the highest similarity to a known gene cluster (22% of genes show similarity). *Cladophialophora immunda* further has clusters with similarity to SMBGCs related to antibiotics, antifungal, and toxins (methylstreptimidone, nivalenol). Nivalenol is a toxin from the group of trichothecene, a compound that seems to play an important role in *Cladophialophora immunda* as this fungus possesses 65 trichothecene efflux pumps.

**Table 1:** Secondary metabolite biosynthesis gene clusters found in *Cladophialophora immunda* with at least one gene similar to a known SMBGC.

Location	antiSMASH type	Most Similar Cluster	Known Cluster
JSEJ01000002.1:442102–524450	cf_putative	*A*. *gypseum* 23%	Huperzine A 7%
JSEJ01000004.1:495938–622933	cf_saccharide	*S*. *usitatus* 6%	Pneumocandin 6%
JSEJ01000024.1:167780–303648	cf_putative	*T*. *tonsurans* 17%	Nivalenol/deoxynivalenol/3-acetyldeoxynivalenol 9%
JSEJ01000031.1:173557–325662	cf_putative	*C*. *psammophila* 40%	Griseofulvin 9%
JSEJ01000071.1:105157–208601	cf_putative	*A*. *terreus* 11%	Isoflavipucine 12%
JSEJ01000084.1:10697–57480	cf_putative	*C*. *psammophila* 46%	1_9-methylstreptimidone 6%
JSEJ01000096.1:7213–92198	cf_putative	*L*. *maculans* 27%	Penicillin 12%
JSEJ01000138.1:62903–96712	t1pks	*C*. *epimyces* 18%	Monacolin K 22%

The first column contains the position of the cluster in *Cladophialophora immunda*, the second column shows the type of the secondary metabolite, the fourth column shows the species with the cluster containing the highest amount of similarity to the cluster found in *Cladophialophora immunda*. The last column shows the secondary metabolite produced by the most similar known SMBGC and the percentage of genes showing similarity.

In the toluene experiment, three backbone genes from three distinct SMBGCs were downregulated, while none were upregulated. CLAIMM 01034, a *β*-ketoacyl synthase, was downregulated by a factor of 17, CLAIMM_06122, an other b-ketoacyl synthase, was downregulated by a factor 5.53, while CLAIMM_09644, an AMP-dependent synthetase and ligase, was downregulated by a factor 6.14.

Due to the presence of trichothecene efflux pumps and a cluster similar to Nivalenol/deoxynivalenol/3-acetyldeoxynivalenol SMBGC, the ability of *Cladophialophora immunda* to produce trichothecene and trichothecene-derived toxins was studied. To this aim homologues of the genes belonging to the core trichothecene clusters of *Fusarium graminearum*, *Fusarium sporotrichoides*, *Trichoderma arundinaceum*, *Trichoderma brevicompactum*, *Stachybotrys chartarum* and *Stachybotrys chloronata* were searched in *Cladophialophora immunda*. Out of the 17 genes found in the core trichothecene cluster of these fungi^68^ (see Supplementary Table S1), 12 were found in the genome of *Cladophialophora immunda*. The presence of Tri4, Tri5 and Tri11 indicates that the fungus may be able to synthesize molecules with the typical trichothecene structure, while the absence of Tri3, Tri7 might hinder *Cladophialophora immunda* from synthesizing trichothecene-derived mycotoxins (See Supplementary Figure S2). Interestingly, one trichothecene pump, CLAIMM_06732, is upregulated by a factor of 23 when *Cladophialophora immunda* is exposed to toluene. Recently a paper on comparative genomics of black yeasts^59^ showed that the trichothecene efflux pump (TEP) underwent an expansion in black yeasts. Our strain of *Cladophialophora immunda* (CBS 110551) exhibits the highest number of trichothecene efflux pumps among the black yeasts studied in this paper. Interestingly in ref. 59, a strain of *Cladophialophora immunda* (CBS 83496) was studied and had a total of 22 annotated TEPs. This large difference might be explained in part by the findings of a recent paper that showed that changes in environment triggers accelerated adaptation by increasing copy number variation in yeast^69^.

We further looked at genes involved in melanin production in *Cladophialophora immunda* with the data provided in ref. 18 for *Exophiala dermatitidis*. *Cladophialophora immunda* possesses three melanin biosynthesis pathways: the DHN-melanin pathway, the DOPA-melanin pathway and the L-tyrosine degradation pathway. Toluene significantly downregulated the DHN-melanin and DOPA-melanin pathways (see Supplementary Table S1). Still hppD, from the L-Tyrosine pathway, an enzyme that converts p-hydoxyphenylpyruvate to homogentisate (HGA), sees its expression increased by a factor of 79 when *Cladophialophora immunda* exposed to toluene. Besides the role of HGA as precursor of pyomelanin, it is also found in the styrene degradation pathway, where HGA is processed to fumarate and acetoacetate before entering the krebs cycle. The enzymes involved in this process are either not significantly regulated (Homogentisate 1,2-dioxygenase, Malelacetoacetate isomerase) or downregulated (Fumarylacetoacetate hydrolase, CLAIMM_05428, log_2_ FC = −2.09 fdr = 3.6 · 10^−6^). As such HGA seems to be rather involved in the melanin than in energy production.

### Sensing

Homologues to genes involved in the stress-activated MAPK pathway from *Saccharomyces cerevisiae*, *Neurospora crassa*, *Schizosaccharomyces pombe* and *Cryptococcus neoformans* were searched in *Cladophialophora immunda* ^70^. Twelve out of 14 genes of the *Schizosaccharomyces pombe* stress-activated MAPK pathway had homologues in *Cladophialophora immunda*, while this proportion dropped to 41% (7/17) for *Saccharomyces cerevisiae*. *Cladophialophora immunda* homologues to Mak1, Mak2, Pap1, Wis1 and Atf1 (*Schizosaccharomyces pombe*) and to Pbs2 (*Saccharomyces cerevisiae*) are all significantly upregulated when toluene is used as sole carbon-source (See Supplementary Table S1).

The use of toluene as sole-carbon source upregulates the AcuK and Snf1 homologues, two genes whose expression is increased under low glucose concentration in fungi^71^. In contrast genes involved in the gluconeogenesis and *β*-oxidation were significantly downregulated.

### Toluene degradation

**Figure 4:**
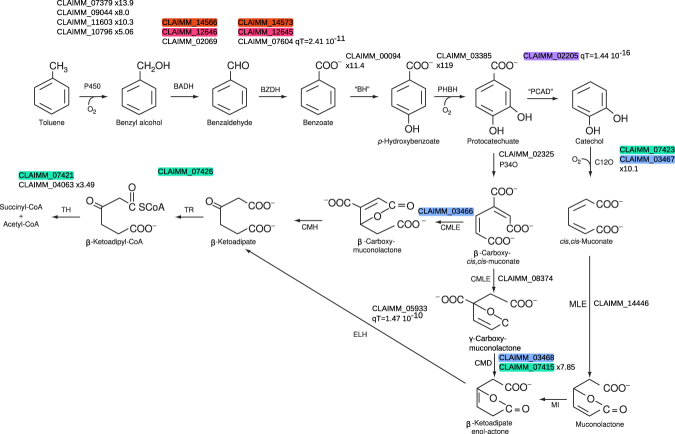
Representation of the pathways related to toluene-degradation found in *Cladophialophora immunda*. For each step in the pathway gene candidates are listed. Information about differential expression and putative horizontal gene transfer are also indicated. The colors highlight genes belonging to the same cluster. Reaction in the teal and brown frame are reported in *P*. *putida mt2* and *P*. *mendocina*, respectively.

The genome of *Cladophialophora immunda* was examined for the presence of genes belonging to the fungal toluene degradation pathways as described in ref. 72 (See Fig. 4 and Supplementary Table S1) by looking at sequence and functional homologies. The initial oxidation of the methyl-group of toluene is catalyzed by a membrane-bound cytochrome P450^73^. *Cladophialophora immunda* has a total of 195 membrane bound Cytochrome P450 (See Supplementary Table S1). Among them, five were upregulated in *Cladophialophora immunda* (see Fig. 4). CLAIMM_00094, one of the five P450 candidates, is probably a benzoate hydroxylase, since it is homologue to *Aspergillus niger* CP53_ASPN (E-value = 0.0), a benzoate hydroxylase.

Benzylalcohol dehydrogenase (aryl-dehydrogenase, BADH) was searched by homology to the aryl-dehydrogenase (xylB) from *P*. *putida mt-2*. The best hit is CLAIMM_14566 (E-value = 3 · 10^−98^), a gene whose homologue in *Fusarium oxysporum* (E-value = 7.2 · 10^−107^) is described as aryl-dehydrogenase. ProteinOrtho predicts that CLAIMM_14566 has two paralogs, CLAIMM_12646 and CLAIMM_02069. None of the BADH was found to be significantly regulated in the toluene experiment.

**Figure 5:**
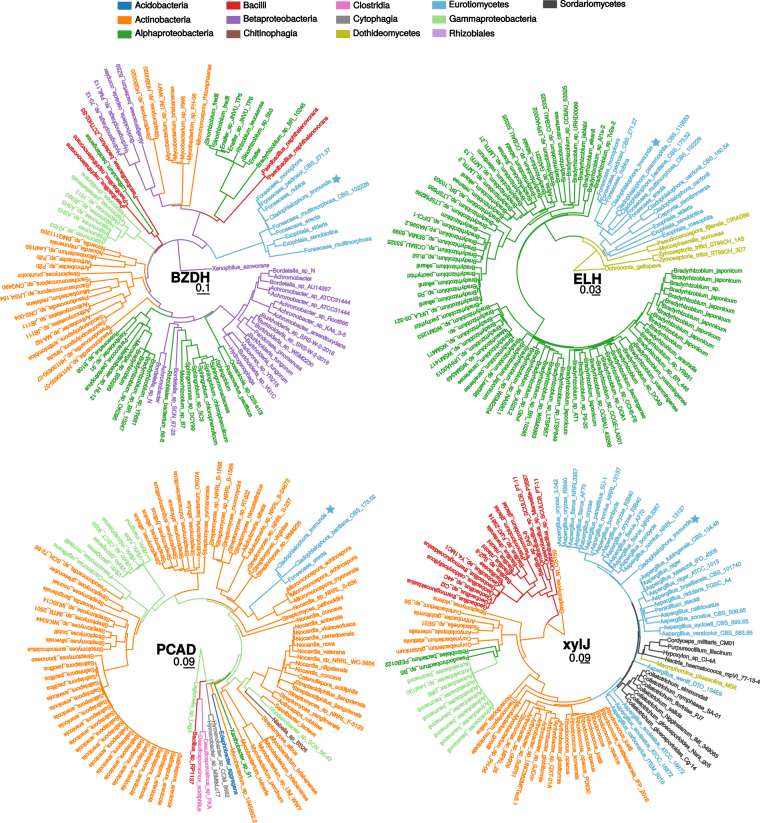
Distance trees of the Blastp hits of CLAIMM_07604 (BZDH), CLAIMM_02205 (PCAD), CLAIMM_05933 (ELH) and CLAIMM_02199 (xylJ) against the nr protein database. The tip labels are colored based on the Class the species belong to. The position of *Cladophialophora immunda* in the tree is marked with a star. Blastp homologues were found in three fungal classes (Eurotiomycetes, Sordariomycetes, Dothideomycetes) and 11 bacterial classes.

Similarly, benzaldehyde dehydrogenase (BZDH) was searched by looking for homologues of xylC in *Cladophialophora immunda*. Three genes, CLAIMM_14573, CLAIMM_12645 and CLAIMM_07604 (E-values 5 · 10^−68^, 1 · 10^−69^ and 1 · 10^−67^, respectively) showed both sequence and functional homologies to the bacterial BZDH. Homologues to CLAIMM_14573 and CLAIMM_12645 were found among our set of genomes solely in *Cladophialophora bantiana* (CLAIMM_14573) and *Fusarium oxysporum* (CLAIMM_12645, CLAIMM_14573) while CLAIMM_07604 had no homologues. In fact, CLAIMM_07604 was signficantly (qT-value 2.41 · 10^−11^) horizontally transferred from the bacterial kingdom to the fungal one. This is further confirmed by the distance tree of its closest homologues found with Blastp against the nr database (Fig. 5). It should be noted that two putative enzymes involved in BADH degradation (CLAIMM_14566, CLAIMM_12646) are located in close genomic vicinity to two of the BZDH-degrading enzymes (CLAIMM_14573, CLAIMM_12645).

Benzoate is converted to *p-*hydroxybenzoate by the benzoate hydroxylase (BH), CLAIMM_00094, which is homologue to *Aspergillus niger* CP53_ASPNG (E-value = 0.0) and is upregulated (x11.4) in the toluene experiment. *p-*hydroxybenzoate is oxidized to protocatechuate by *p-*hydroxybenzoate hydroxylase (PHBH). In *Cladophialophora immunda*, five genes (CLAIMM_02622 CLAIMM_03385 CLAIMM_08446 CLAIMM_09063 CLAIMM_11189) have the PF07976 domains, which was shown to be involved in the hydroxylation of p-hydroxybenzoate^74^. Among those genes only CLAIMM_03385 was upregulated (x119) when *Cladophialophora immunda* was exposed to toluene.

Protocatechuate is then degraded by two distinct pathways to *β*-ketoadipate. In one branch, the aromatic ring is opened by the dioxygenase (P340), CLAIMM_02325, an homologue of CCT72286 (*Fusarium fujikuroi*, E-value = 5 · 10^−127^). *β*-carboxy-cis,cis-muconate is then processed by *β*-carboxy-*cis*,*cis*-muconate lactonizing enzyme (CMLE). *Cladophialophora immunda* has one CMLE homologue to *Neurospora crassa* CMLE (CLAIMM_03466, E-value = 4 · 10^−176^) and one homologue to *Acinetobacter baylyi* CMLE (CLAIMM_08374, E-value = 2 · 10^−93^). Both enzymes have the same substrate, but delivers *β* or *γ* -carboxy-muconolactone, respectively. *β* -carboxy-muconolactone is processed by *β* -carboxy-muconolactone hydrolase (CMH), an enzyme for which no candidate could be found in fungi. However *γ* -carboxy-muconolactone is processed by carboxy-muconolactone decarboxylase (CMD) to *β* -ketoadipate enol-actone. A total of 13 genes in *Cladophialophora immunda* have a CMD-like protein domain (IPR003779), with CLAIMM_07415 being upregulated (x7.85) in the toluene experiment.

In the second branch of the *β* -ketoadipate pathway, PCAD converts protocatechuate to catechol. *Cladophialophora immunda* contains 4 genes (CLAIMM_04693, CLAIMM_14616, CLAIMM_09412, CLAIMM_02205) belonging to the UbiD decarboxylase family (IPR002830), whose members are involved among others in the decarboxylation of protocatechuate^75^. Based on HGTfinder, CLAIMM_02205 is of bacterial origin *Cladophialophora immunda* (qT-value = 1.44 · 10^−16^, see Fig. 5). The subsequent oxidation of catechol by catechol 1,2-dioxygenase (C12O, × 10.1 in toluene, CLAIMM_03467, homologue to *Aspergillus niger* An13g02000 E-value = 2 · 10^−145^) opens the aromatic ring and converts it to *cis*,*cis*-muconate. Six other genes in *Cladophialophora immunda* have catechol 1,2-dioxygenase activity (See Supplementary Table S1) but none of them are significantly regulated. *Cis*,*cis*-muconate lactonizing enzymes (CLAIMM_14446) (E-value 4 · 10^−104^ MLE *Trichosporon cutaneum*) yields muconolactone that is processed by muconolactone isomerase (MI). While this enzyme has been reported to be present in fungi, no corresponding fungal sequence could be found in the litterature. *β* -ketoadipate-enol-actone hydrolase (CLAIMM_05933, ELH from *Cladophialophora carrionii*, E-value = 9 · 10^−134^), is of bacterial origin (qT = 1.47 · 10^−10^, see Fig. 5) and delivers *β* -ketoadipate.

*β*-ketoadipate is then processed to *β*-ketoadipyl-CoA by *β*-ketoadipate succinyl-CoA transferase (CLAIMM_7426, E-value 4 · 10^−56^ from *P*. *putida* pcaJ). *β*-ketoadipate succinyl-CoA is converted to Succinyl-CoA and Acetyl-CoA by *β*-ketoadipyl-CoA thiolase. In *Cladophialophora immunda* 10 genes have a thiolase active site, among which one is upregulated (CLAIMM_04063 × 3.49) and one is located close to genes all involved in the degradation of toluene (CLAIMM_07421).

Besides the genes involved in the toluene degradation, the presence of functional and sequence homologues of bacterial genes responsible for toluene degradation was investigated. xylL is involved in the conversion of toluate cis-dihydrodiol dehydrogenase in *P*. *putida* ^72^. It has 33% sequence similarity to CLAIMM_00812 (Blastp E-value = 2 · 10^−32^), similar length and protein domains (PS00061, PR00080, PR00081, SSF51735, PF00106, IPR002347, IPR020904, IPR016040) (See Supplementary Figure S3). Similarly xylJ, 2-hydroxypent-2,4-dienoate hydratase, shows 34% sequence similarity to CLAIMM_02199, as well as identical domain annotation (IPR011234, SSF56529, G3D 3.90.850.10). CLAIMM_02199 is interestingly also classified as horizontally transferred by HGTfinder (qT = 1.1 · 10^−5^, see Fig. 5) and is located close to CLAIMM_02205 (PCAD).

CLAIMM_07216 is an homologue of yeast YNR064C, a gene that belongs to the xylF family of esterase, whose members are responsible for the conversion of 2-hydroxymuconic semialdehyde to 2-oxopent-4-eneoate (See Supplementary Figure S3).

In *Pseudomonas mendocina*, *pcuAB* is a dimer responsible for the oxydation of *p*-cresol to *p*-Hydroxy-benzaldehyde. CLAIMM_11938, a gene upregulated (x18.03) upon toluene exposure, is homologue to PcuB (E-value 3 · 10^−113^) and harbours the same IPR annotations. Interestingly members of the *Cladophialophora* genus were reported to degrade p-cresol^76^. *pcuC* is homolog to CLAIMM_09141 (E-value = 4 · 10^−73^) and CLAIMM_08951 (E-value 8 · 10^−73^), the latter having probably been horizontally transferred from bacteria to fungi (qT-value = 3 · 10^−3^). *pcuC* is responsible for the oxydation of *p*-Hydroxy-benzaldehyde to *p*-Hydroxy-benzoate.

A look at the genomic organization of the genes involved in the toluene degradation indicates that 13 of them are grouped into 5 clusters. The first two clusters contain four genes corresponding to BADH and BZDH. Seven genes involved in catechol and protocatechuate degradation are distributed into two clusters respectively (see Fig. 4). The last cluster contains the genes PCAD and the homologue of xylJ, which were both horizontally transferred (Figs 4 and 5).

## Discussion

In this study, for the first time, the transcriptome of a fungus growing on toluene^12^ was sequenced. With the help of the transcriptomic data and a comparative genomics analysis, genes previously reported to play a role in metabolizing toluene in other fungi and bacteria^72^ were identified and their expression levels during the toluene exposure were assessed. For all but two enzymes in the fungal toluene-degrading pathway described in ref. 72, at least one orthologue was found. The genomic organization of these coding loci in *Cladophialophora immunda* is highly interesting since five gene clusters were identified. While functional clusters are common in fungi^77^, it is the first time that this genomic organization is reported for clusters of genes related to hydrocarbon degradation.

The comparison of the fungal and bacterial toluene-degradation pathways allowed to identify 8 genes in *Cladophialophora immunda* that are responsible for toluene degradation steps in *P*. *putida* ^78^ and *P*. *mendocina* ^72^. Among those 8 genes, four were classified as horizontally transferred from bacteria by HGTfinder, further underlining the close interplay between bacteria and the studied *Cladophialophora*. While HGTs are common in fungi, they were only recently reported in the group of black yeasts, i.e. for *Exophiala dermatitidis* ^18^. Especially interesting are CLAIMM_02205 (PCAD) and CLAIMM_02199 (xylJ) (See Fig. 5). CLAIMM_02205 is the only gene that converts protocatechuate to catechol while CLAIMM_02199 is the only gene that catalyzes the hydration of 2-oxopent-4-eneoate into 4-hydroxy-2-oxovalerate. The fact that the homologues to bacterial enzymes partially cover additional toluene-degradation pathway might be an evidence that in a toluene-contaminated environment *Cladophialophora immunda* and bacterial species are cooperating in metabolizing toluene, similar to what was shown for polyaromatic hydrocarbons^79^.

The genomic analysis of significantly enriched protein domains lead to some interesting observations about the ecology of *Cladophialophora immunda*. The fact that proteins involved in complex carbohydrates degradation and sugar transport are overrepresented might suggest that the fungus evolved in an oligotrophic and polluted environment. The overrepresentation of trichothecene efflux pumps and the upregulation of one them by a factor 23 in the toluene experiment, might signal that this secretion system is involved in the excretion of toluene or secondary metabolites.

Despite the ability of *Cladophialophora immunda* to degrade toluene, its presence drives the fungus into an energy-saving state. Core processes like cellular amid metabolism, cellular respiration, energy-coupled proton transport, organic substance metabolism are negatively affected by the xenobiotic. Translation is one of the most enriched GO term (fdr = 1.17 · 10^−46^) in the set of downregulated genes. This fits well with the repression of 17 tRNAs during toluene exposure. In contrast, snoRNAs, which are responsible for the rRNA maturation^80^, represent the only type of basal ncRNAs that are significantly upregulated in the toluene experiment. In *Escherichia coli*, toluene triggers the rapid disaggregation of ribosomes^81^. Here the increased expression of snoRNAs might be interpreted as response to the destabilizing effect of toluene on the maturation process of rRNAs.

Chaperones HSP20, HSP40 and HSP70, which are markers of cellular stress^82^, are either highly expressed or highly upregulated in the toluene -experiment. This fact, together with the upregulation of genes involved in the transport of misfolded proteins, might indicate that toluene is having a negative impact on the protein-folding process, as recently reported in prokaryotes^83^. Toluene exposure also leads to the overexpression of genes harboring protein domains linked to DNA repair, like ku70/ku80 and rad14, indicating that the fungal DNA is being damaged. This is in line with the recognized mutagenic properties of aromatic and other poly-aromatic compounds^84^.

Toluene further triggers the expression of antioxidants as well as genes involved in cell detoxification, like Glyoxylase II, Glutathione S-transferase, Ascorbate peroxidase and Carotenoid oxygenase. HppD, a key enzyme in the production of Pyomelanin is upregulated by a factor 79 when *Cladophialophora immunda* is exposed to toluene. This melanin type was shown to be overexpressed when *Exophiala dermatitidis* is grown on skin^20^ and to protect against hydrogen peroxide stress^85^ in the soil-borne bacteria *Ralstonia solanacearum*.

Six genes belonging to the stress-induced MAPK pathway in fungi are upregulated in the toluene experiment. This signaling pathway is related to various kind of stress, among others oxidative stress^70^. This, together with the damaged-DNA repair response and melanin production, suggests that toluene is causing an oxidative burden on *Cladophialophora immunda*, something previously reported in prokaryotes^64^.

In conclusion, the thorough genome annotation of *Cladophialophora immunda* paired with the transcriptome data allows us to get a better insight into the mechanisms used by this fungus to protect itself from toluene and degrade it. Even though this fungus is one of the most efficient toluene degrader^12^, this xenobiotic is sensed by *Cladophialophora immunda* as a toxic compound that triggers a strong stress reaction and the downregulation of various basal metabolisms.

## Electronic supplementary material

Supplementary Figures

Dataset 1
