# Supplementary material for: Genomic and transcriptomic analysis of the toluene degrading black yeast Cladophialophora immunda
Source: Sci Rep. 2017 Sep 12;7:11436. doi: 10.1038/s41598-017-11807-8 (PMC5595782; doi:10.1038/s41598-017-11807-8)
Supplement: Supplementary file 1 — Supplementary Figures [file 41598_2017_11807_MOESM1_ESM.pdf]

Genomic and transcriptomic analysis of the toluene degrading black yeast *Cladophialophora immunda*

Barbara Blasi, Hakim Tafer, Christina Kustor, Caroline Poyntner, Ksenija Lopandic, Katja Sterflinger

Department of Biotechnology, VIBT-EQ Extremophile Center, University of Natural Resources and Life Sciences, 1190 Vienna, Austria.

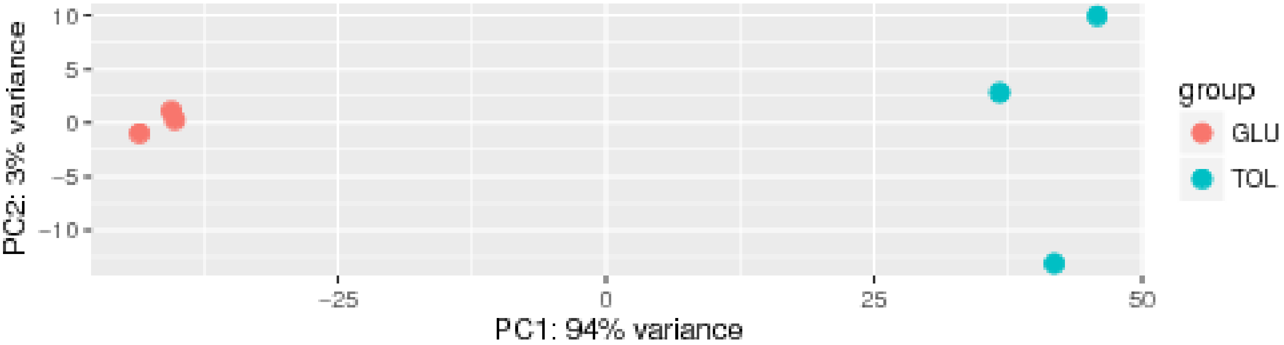

Supplementary Figure S1

Principal component analysis of the expression pattern between the toluene (cyan) and glucose (red) experiments. For each condition,each dot correspond to one replicate.

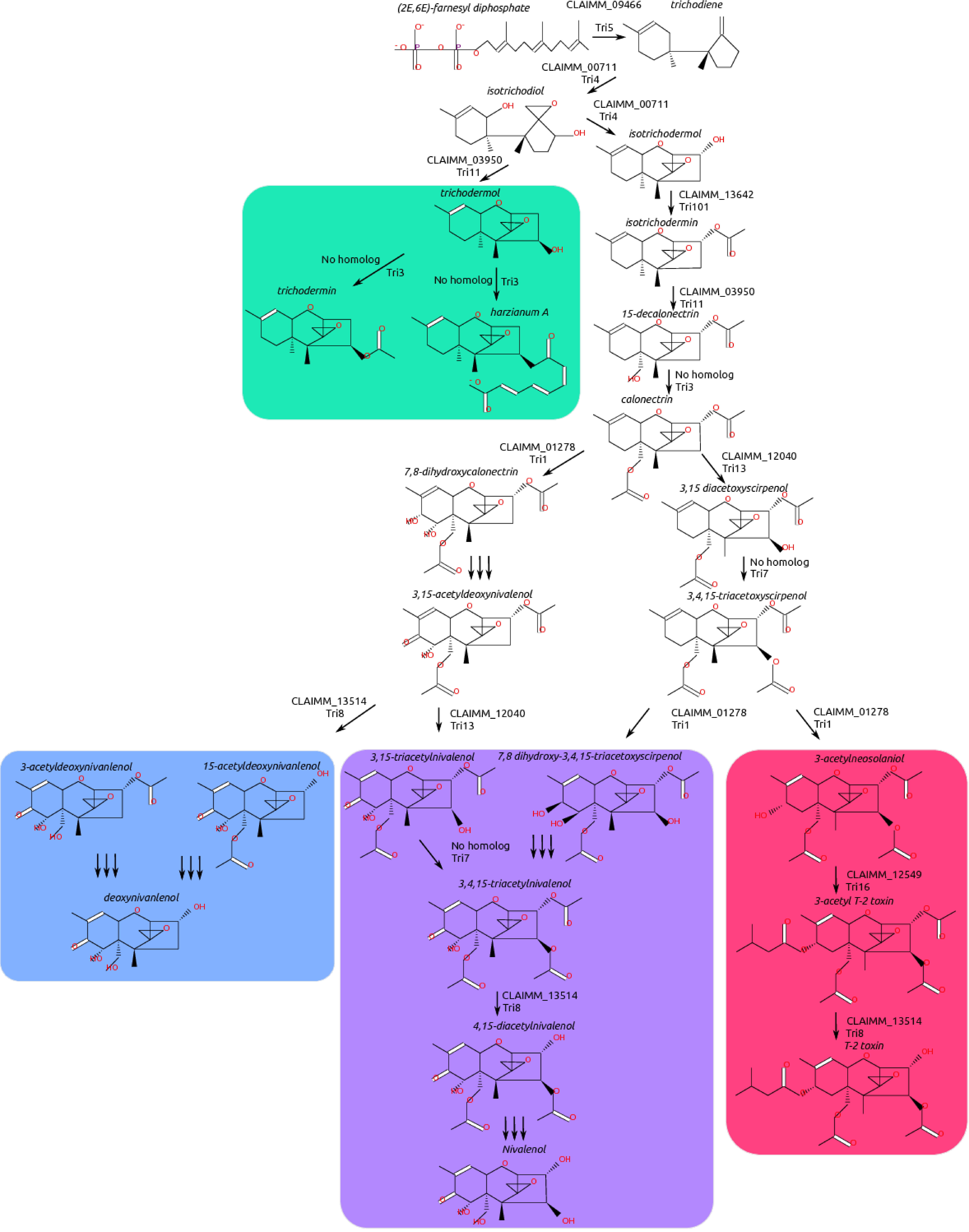

Supplementary Figure 2: Superpathway of trichothecene biosynthesis from Metacyc. Official gene names and the corresponding C. Immunda names are shown for each steps. Each colored box represent a subpathway leading to a different toxin.

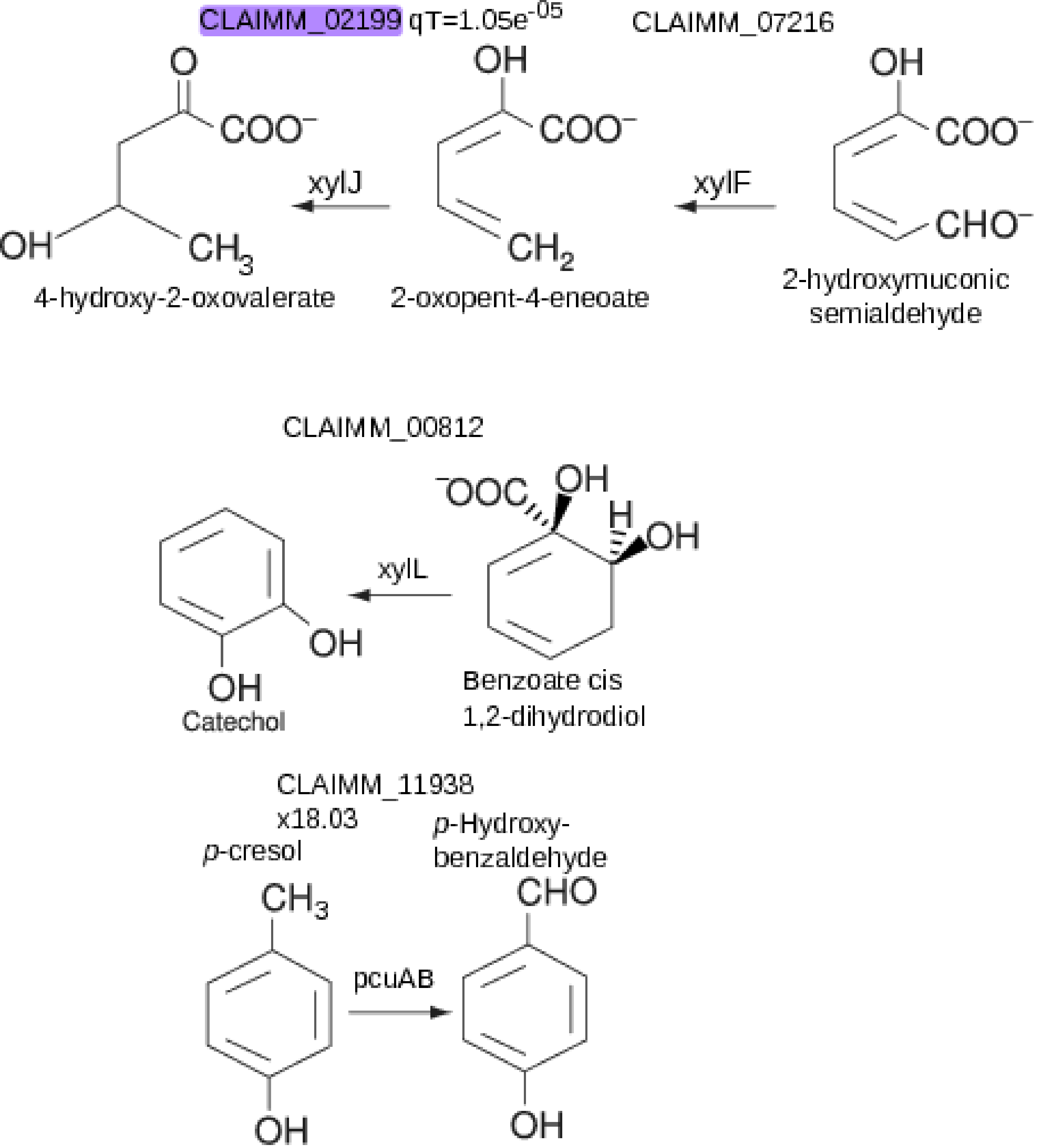

Supplementary Figure S3:

Part of the bacterial toluene degrading pathways, for which homologous enzymes were found in C. immunda.
